# An e-Delphi Study to Develop and Validate a Delirium Caregiving Self-Efficacy Scale

**DOI:** 10.1093/geroni/igaf122.1569

**Published:** 2025-12-31

**Authors:** Shih-Yin Lin, Zachary Kunicki, Abraham Brody, Donna Fick

**Affiliations:** New York University Rory Meyers College of Nursing, New York, New York, United States; Warren Alpert Medical of Brown University, Providence, Rhode Island, United States; New York University Rory Meyers College of Nursing, New York, New York, United States; The Pennsylvania State University, University Park, Pennsylvania, United States

## Abstract

Delirium is an acute neurocognitive disorder that commonly occurs in older adults, especially those with dementia. Multicomponent non-pharmacological interventions are the most effective for delirium prevention. While caregiver-administered multicomponent interventions show promise, adherence to different components varies. Self-efficacy may play a role in determining adherence. To explore this relationship, a validated delirium caregiving self-efficacy scale is needed. This study aimed to develop such a scale and establish its content validity using an e-Delphi expert panel. First, a rapid review using the NIDUS Delirium Bibliography identified delirium experts (corresponding authors of relevant publications) to invite and key domains of multicomponent interventions to guide the development of candidate scale items. Two rounds of e-Delphi surveys followed, during which experts rated the relevance, clarity, and importance of candidate items using Likert-type scales. Content validity was assessed using the content validity index (CVI, cutoff: 0.8) and content validity ratio (CVR, cutoff: 0.49). The rapid review identified 16 domains—leading to the development of 167 candidate items, and 70 experts. Twenty identified experts participated, with 18 completing both rounds. In Round 1, 61 items (36.5%) met both CVI and CVR cutoffs and were retained. Experts also suggested 25 new items. In Round 2, four of the 25 new items met the CVI and CVR criteria, resulting in a final 65-item scale. These findings support the content validity of the 65-item scale. Future research should refine it through cognitive interviews with caregivers to ensure comprehension and conduct item reduction analysis to create a more concise measure.

